# A convolutional neural network-based regression model to infer the epigenetic crosstalk responsible for CG methylation patterns

**DOI:** 10.1186/s12859-021-04272-8

**Published:** 2021-06-23

**Authors:** Wan Kin Au Yeung, Osamu Maruyama, Hiroyuki Sasaki

**Affiliations:** 1 Division of Epigenomics and Development, Medical Institute of Bioregulation, Kyushu University, Fukuoka, 812-8582 Japan; 2 Faculty of Design, Kyushu University, Fukuoka, 815-0032 Japan

**Keywords:** DNA methylation, Histone modification, Epigenetic crosstalk, Convolutional neural network, Oocyte, Stem cell

## Abstract

**Background:**

Epigenetic modifications, including CG methylation (a major form of DNA methylation) and histone modifications, interact with each other to shape their genomic distribution patterns. However, the entire picture of the epigenetic crosstalk regulating the CG methylation pattern is unknown especially in cells that are available only in a limited number, such as mammalian oocytes. Most machine learning approaches developed so far aim at finding DNA sequences responsible for the CG methylation patterns and were not tailored for studying the epigenetic crosstalk.

**Results:**

We built a machine learning model named epiNet to predict CG methylation patterns based on other epigenetic features, such as histone modifications, but not DNA sequence. Using epiNet, we identified biologically relevant epigenetic crosstalk between histone H3K36me3, H3K4me3, and CG methylation in mouse oocytes. This model also predicted the altered CG methylation pattern of mutant oocytes having perturbed histone modification, was applicable to cross-species prediction of the CG methylation pattern of human oocytes, and identified the epigenetic crosstalk potentially important in other cell types.

**Conclusions:**

Our findings provide insight into the epigenetic crosstalk regulating the CG methylation pattern in mammalian oocytes and other cells. The use of epiNet should help to design or complement biological experiments in epigenetics studies.

**Supplementary Information:**

The online version contains supplementary material available at 10.1186/s12859-021-04272-8.

## Background

Epigenetic modifications, including DNA methylation and histone modifications, play important roles in embryonic development in part through the regulation of gene transcription [1]. Increasing evidence suggests that the epigenetic modifications interact with each other to shape their distribution patterns [2]. In mammals, CG methylation, a major form of DNA methylation, undergoes global reprogramming during germ-cell development. Then, in the postnatal ovary, an oocyte-specific CG methylation pattern is established by de novo DNA methyltransferase DNMT3A [3]. In vitro studies revealed that DNMT3A contains protein domains that potentially interact with histone modifications and regulate recruitment of this protein to target loci [4, 5]. Indeed, reverse genetics approaches began to reveal the impact of histone modifications, such as H3K36me3, on the establishment of CG methylation in mouse oocytes [6], but the entire picture of the epigenetic crosstalk regulating CG methylation patterns is unknown. This is partly due to the scarcity of oocyte samples available for molecular studies and the high cost required for a genome-wide epigenetic modification analysis. Previous studies showed that machine learning approaches are useful for predicting CG methylation patterns based on limited measurable features (mainly DNA sequence information) and thus may complement biological experiments [7–10]. However, most of the existing approaches aim at finding DNA sequences responsible for the CG methylation patterns and were not tailored for studying the epigenetic crosstalk.

In this study, we built a machine learning model named epiNet to predict CG methylation patterns based on other epigenetic features. Using epiNet, we identified biologically relevant epigenetic crosstalk between histone H3K36me3, H3K4me3, and CG methylation in mouse oocytes. This model also predicted the altered CG methylation pattern of mutant oocytes having perturbed histone modification and was applicable to cross-species prediction of the CG methylation pattern of human oocytes. It also identified the epigenetic crosstalk potentially important in other cell types. The use of epiNet should help to design or complement biological experiments in epigenetics studies.

## Results

### Outline of epiNet: prediction of CG methylation patterns based on other epigenetic features

**Fig. 1:**
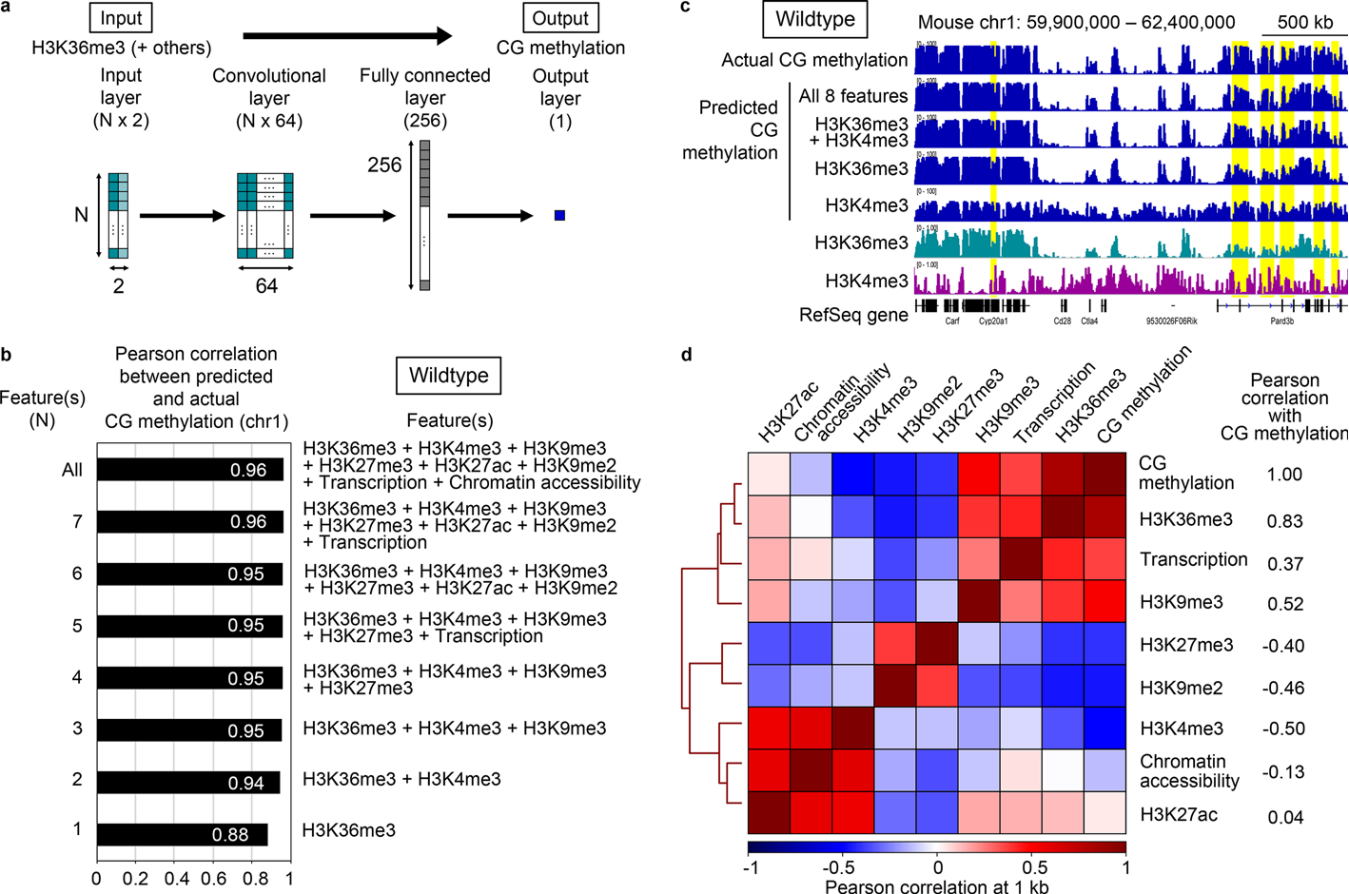
Building epiNet models to predict CG methylation patterns based on other epigenetic features. **a** The epiNet model. Input FPKM values were transformed by one convolutional layer and one fully connected layer to predict the CG methylation levels of 1-kb bins (see “Methods” section). The shape of each layer is indicated within bracket. N: Number of input features. **b** The prediction of the CG methylation pattern of mouse FGOs based on varying numbers of input features. For each number of features (N = 1–8), the feature combination that showed the best correlation between the predicted and actual CG methylation patterns is shown. **c** A representative genome browser shot showing the predicted CG methylation patterns. The actual CG methylation, H3K36me3 and H3K4me3 patterns are shown for comparison. Genomic regions in which prediction was improved by the addition of the H3K4me3 data are highlighted in yellow. RefSeq genes are shown at the bottom. **d** Pairwise correlations between the actual data of all epigenetic features. Pearson correlation coefficients between the CG methylation pattern and the distributions of the respective features are indicated on the right

To aid in the identification of epigenetic features regulating CG methylation patterns, we developed a convolutional neural network-based regression model named epiNet, which predicts CG methylation patterns based on other available genome-wide epigenetic features. Contrary to the previous machine learning approaches, epiNet does not use DNA sequences as input, in an attempt to focus on the interactions between the epigenetic features. epiNet consisted of four layers, including one convolutional and one fully connected layer, and built a model for the prediction of CG methylation levels of 1-kilobase (kb) bins (genomic segments) based on the fragment per kb per million mapped reads (FPKM) values of input data (Fig. 1a and “Methods” section). Datasets of the following features from fully-grown mouse oocytes (FGOs) or metaphase II oocytes were used as input: six histone modifications, chromatin accessibility, and transcription [6, 11–17] (Additional file 1: Table S1). To incorporate the effect of the epigenetic state of the neighboring regions, the mean of FPKM values of 10 nearby (5 upstream and 5 downstream) bins was also used (Additional file 1: Fig. S1a, b). CG methylation data were obtained from whole genome bisulfite sequencing (WGBS) [6, 12].

### Prediction of the epigenetic crosstalk responsible for CG methylation patterns by epiNet models

To examine to what extent the features cooperate to impact CG methylation, all possible combinations (n = 255) of the eight features were respectively used as input. We set aside datasets from chromosome 1 for performance test and used those from all other chromosomes for training and validation. As a result, a combination of all eight features (N = 8) gave the highest predictive power (correlation with the actual CG methylation data (R) = 0.96) (Fig. 1b, c). We then sought combinations of a smaller number of features that achieved good performance and found that even H3K36me3 alone had high predictive power (R = 0.88) (Fig. 1b, c). Our model based on H3K36me3 outperformed the baseline method of linear regression (R = 0.83) (Additional file 1: Fig. S1b), especially at gene bodies and intergenic regions, but not at CpG islands (Additional file 1: Fig. S1c). Importantly, when a randomly shuffled H3K36me3 dataset was used, there was no correlation with the actual data (R = 0.00) (Additional file 1: Table S2). A combination of H3K36me3 and H3K4me3 showed a higher correlation (R = 0.94), which was close to the value achieved by all of the features (R = 0.96) (Fig. 1b, c). This is consistent with the fact that, in FGOs, the genomic distribution of CG methylation shows the highest correlation with that of H3K36me3 (R = 0.83) and highest anti-correlation with that of H3K4me3 (R = − 0.50) (Fig. 1d). The predicted CG methylation patterns showed that the addition of H3K4me3 greatly improved the prediction at CpG islands (Additional file 1: Fig. S1d), which are rich in H3K4me3 [18]. Consistent with this, H3K4me3 alone showed a high predictive power at CpG islands (R = 0.85) (Additional file 1: Fig. S1c) but not in the entire genome (R = 0.58) (Additional file 1: Table S2). The further addition of H3K9me3, H3K9me2 and H3K27me3, which were either correlated or anti-correlated with CG methylation (R = 0.52, − 0.46 and − 0.40), slightly improved the prediction (R = 0.95) (Fig. 1b).

**Fig. 2:**
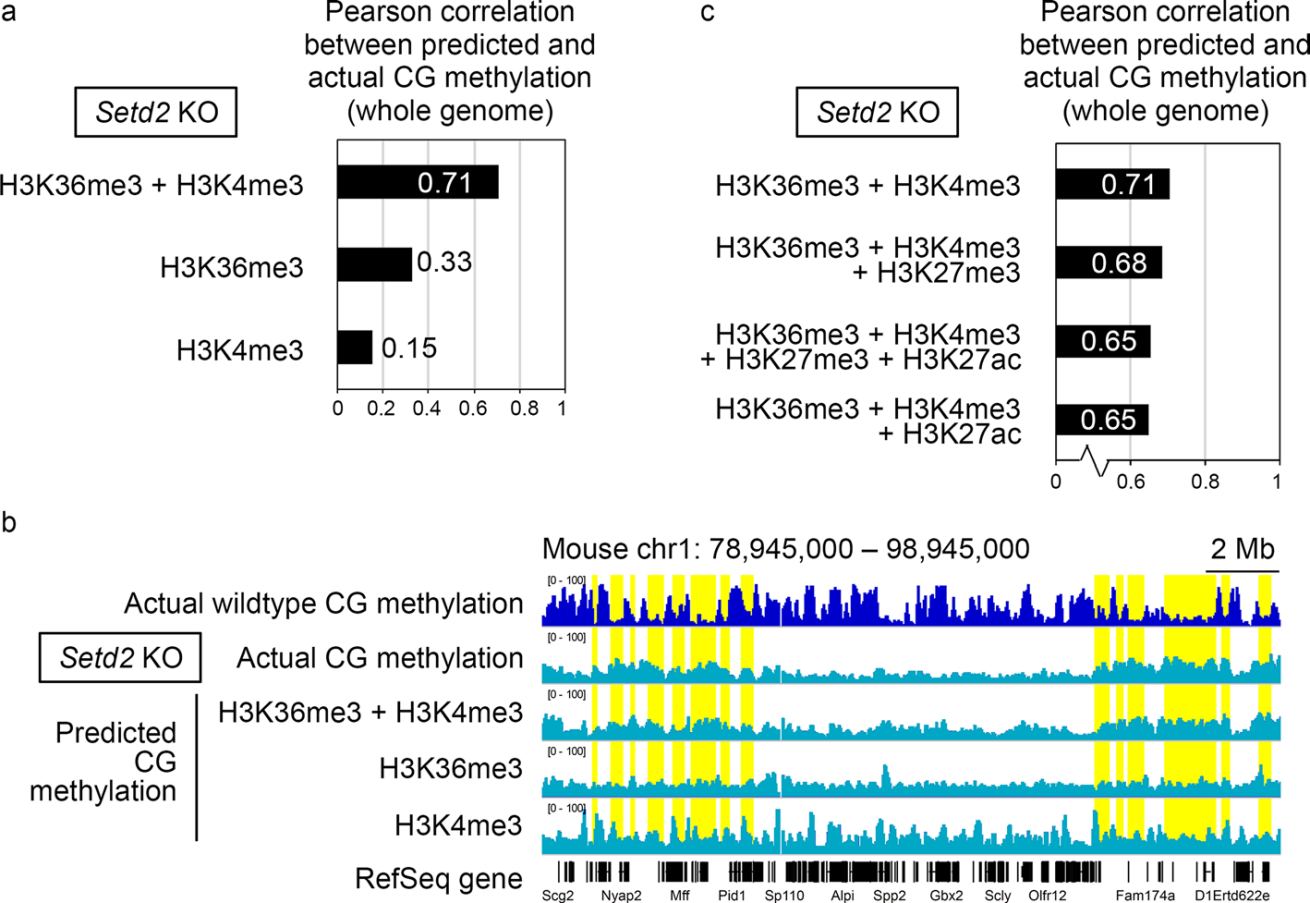
Application of epiNet models to predict the CG methylation pattern of *Setd2* KO FGOs. **a** Pearson correlation coefficients between the predicted and actual CG methylation patterns of *Setd2* KO FGOs. epiNet models trained with the data of the indicated feature(s) from wildtype FGOs were used to predict the CG methylation pattern for the input data of the same feature(s) from *Setd2* KO FGOs. The *Setd2* KO data were from the entire genome. **b** A representative genome browser shot showing the predicted CG methylation patterns of *Setd2* KO FGOs. The actual CG methylation patterns of wildtype and *Setd2* KO FGOs are shown for comparison. Genomic regions showing CG methylation gains are highlighted in yellow. RefSeq genes are indicated at the bottom. **c** The effect of additional features on the prediction of the CG methylation pattern of *Setd2* KO FGOs. The details are the same as in **a**

Are H3K36me3 and H3K4me3 biologically relevant to CG methylation? Previous gene knockout (KO) studies on histone modification enzymes showed that the depletion of H3K36me3 (by *Setd2* KO, see below) causes a genome-wide loss of CG methylation (with occasional local gains) in oocytes [6] and that the depletion of H3K4me3 (by *Mll2* KO) causes local changes in CG methylation (more losses than gains) [11] (Additional file 1: Fig. S2) (see also Fig. 2b for H3K36me3 depletion). These results suggest that the two features, which showed the highest contribution to the in silico prediction of the CG methylation pattern, are biologically relevant. This in turn suggests that prediction using epiNet may aid experimental biologists in tasks such as the selection of KO targets among the epigenetic modification enzyme genes.

### Prediction of the altered CG methylation patterns of mutant oocytes having perturbed histone modifications

We next investigated whether epiNet, when trained with the wildtype dataset, would predict the CG methylation changes upon perturbation of an epigenetic feature. Only one previous study, an oocyte-specific KO of *Setd2* (the only H3K36me3 enzyme) [6] provided a dataset that could be used to answer this question (Additional file 1: Table S1). In this analysis, we used 50-kb bins due to the resolution limit of the data. When H3K36me3 and H3K4me3 from *Setd2* KO FGOs were used as input, we observed a moderate correlation between the predicted and actual CG methylation patterns (R = 0.71). However, the performance dropped when H3K36me3 (R = 0.33) or H3K4me3 alone was used (R = 0.15) (Fig. 2a). A closer examination of the predicted pattern revealed that a combination of H3K36me3 and H3K4me3 reproduced not only the global loss, but also the local gains, of CG methylation in *Setd2* KO FGOs (Fig. 2b). Although the local gains in CG methylation coincided with the reductions in H3K4me3, H3K27me3 and H3K27ac in KO FGOs [6], the inclusion of the H3K27me3 and H3K27ac data caused a deterioration in predictive performance (R = 0.65–0.68) (Fig. 2c).

### Cross-species application of epiNet models to human oocytes

**Fig. 3:**
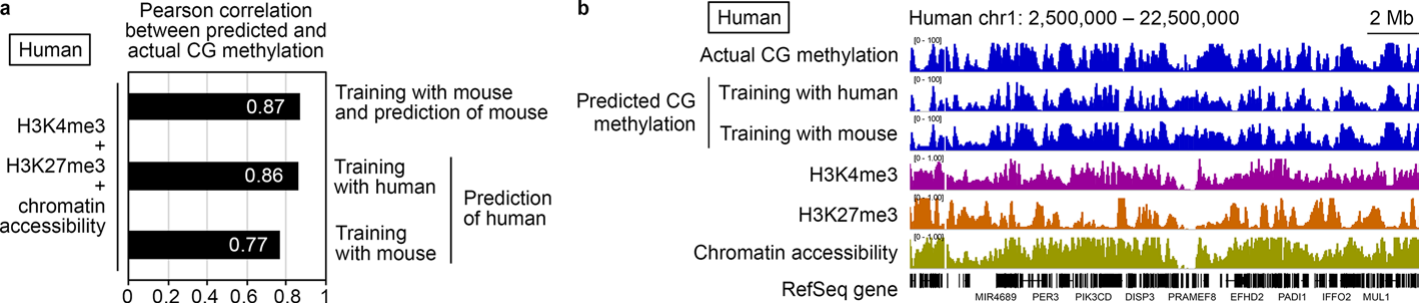
Cross-species application of epiNet models to human oocytes. **a** Pearson correlation coefficients between the predicted and actual CG methylation patterns of human FGOs. The features used to predict the CG methylation pattern were H3K4me3, H3K27me3 and chromatin accessibility. The species from which the training and test data originated are indicated. When training and testing were performed in the same species, the test data were from chromosome 1 and training and validation data were from the rest of the chromosomes. For cross-species testing, H3K4me3, H3K27me3 and chromatin accessibility data from all mouse chromosomes other than chromosome 1 were used to build an epiNet model. Then, H3K4me3, H3K27me3 and chromatin accessibility data from the entire human genome were used to predict the human CG methylation pattern. **b** A representative genome browser shot showing predicted CG methylation patterns of human FGOs. The actual CG methylation, H3K4me3 enrichment, H3K27me3 enrichment, and chromatin accessibility of human FGOs are shown for comparison. RefSeq genes are indicated at the bottom

We next examined whether epiNet could be used to predict the CG methylation pattern of human oocytes, which are precious and not easily accessible. The model would be especially useful if it performs reasonably well based on a limited number of epigenetic features or a limited amount of the feature data. Other than CG methylation data [19], only H3K4me3, H3K27me3 and chromatin accessibility data were available from human FGOs [20] (Additional file 1: Table S1). We first tested whether the same features of mouse FGOs could predict the mouse CG methylation pattern and observed good performance (R = 0.87) (Fig. 3a, b). We then used the human dataset for testing (chromosome 1) and training/validation (all other chromosomes) and observed similarly good predictive performance (R = 0.86). Notably, when the human dataset (the entire genome) were applied to an epiNet model trained with the mouse dataset (chromosomes other than chromosome 1), we still observed a reasonably high correlation between the predicted and actual CG methylation patterns (R = 0.77) (Fig. 3a, b). This suggests that the cross-species application of an epiNet model is possible within the mammalian class.

### Application of epiNet to other cell types

**Fig. 4:**
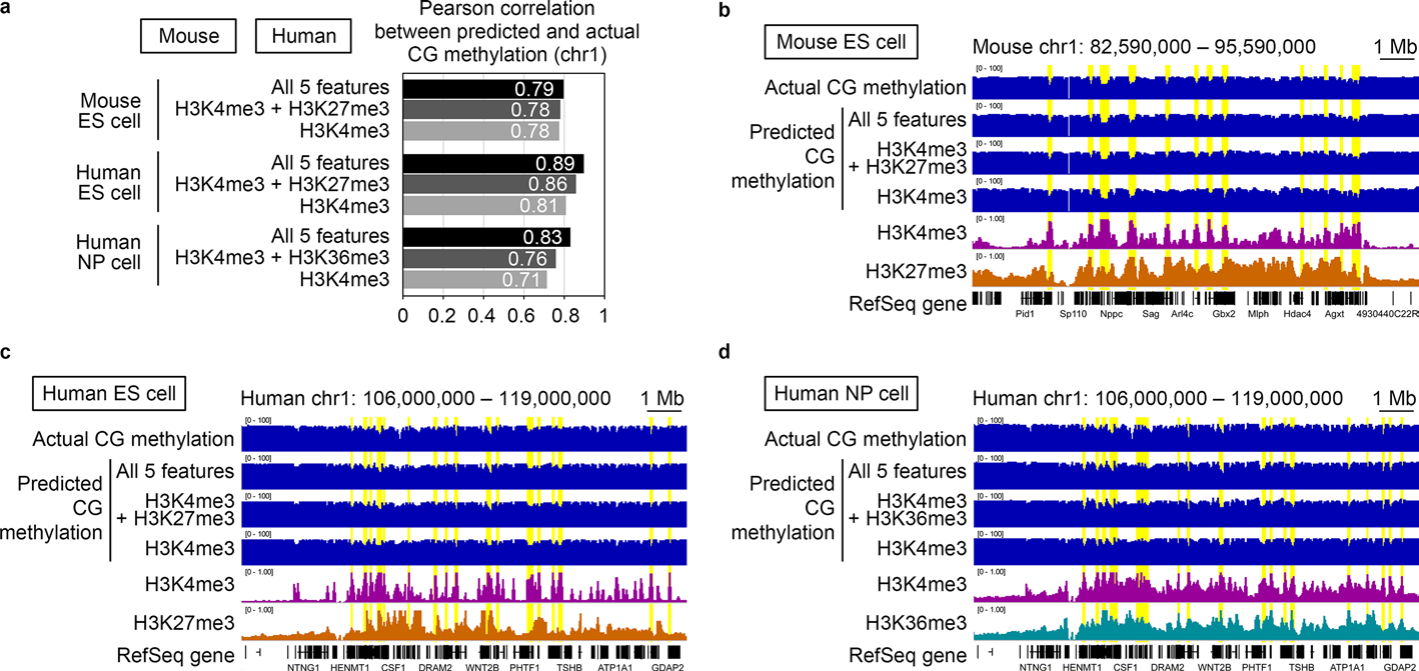
Application of epiNet to other cell types. **a** The prediction of the CG methylation pattern of mouse embryonic stem (ES) cells, human ES cells, and human neuronal progenitor (NP) cells based on varying numbers of input features. For each number of features (N = 1, 2 or 5), the feature combination that showed the best correlation between the predicted and actual CG methylation patterns is shown. **b**–**d** Representative genome browser shots showing the predicted CG methylation patterns of mouse ES cells (**b**), human ES cells (**c**) and human NP cells (**d**). Together with the actual CG methylation pattern, the patterns of the two histone modifications responsible for the best prediction of CG methylation (H3K4me3 and either H3K27me3 or H3K36me3) are shown for comparison. Genomic regions in which H3K4me3 enrichment coincides with a local reduction of CG methylation are highlighted in yellow. RefSeq genes are shown at the bottom

We then investigated whether epiNet can be applied to cells other than oocytes. We focused on three cell types: mouse embryonic stem cells, human embryonic stem cells, and human neuronal progenitor cells, all of which have de novo CG methylation activity (and actively create a CG methylation pattern) as oocytes [21, 22]. Importantly, a common set of data comprising CG methylation and five histone modifications was available for these cell types (Additional file 1: Table S1) [23–25]. When all possible combinations (n = 63) of the five features (histone modifications) were respectively used as input, we found that a combination of all five features (N = 5) gave the highest correlation with the actual CG methylation data in these cell types (R = 0.79–0.89) (Fig. 4a, b, c, d). We then found that even H3K4me3 alone had a high predictive power (R = 0.71–0.81). This contrasts with the highest performance achieved with H3K36me3 in oocytes but is consistent with the fact that, in all three cell types, the genomic distribution of CG methylation shows the highest anti-correlation with that of H3K4me3 (R = − 0.74 to − 0.60) (Additional file 1: Table S3). A combination of H3K4me3 and the best second feature (either H3K27me3 or H3K36me3, depending on the cell type) showed a higher correlation (R = 0.76–0.86), which was close to the value achieved by all of the features.

Are these features biologically relevant to CG methylation in these cell types? In mouse embryonic stem cells, a H3K4me3 depletion (by *Mll2* KO) and a H3K27me3 depletion (by *Eed* KO) respectively caused a genome-wide gain and redistribution of CG methylation [26, 27]. Thus, the two features showing the highest contribution to the in silico prediction were indeed biologically relevant. While the biological relevance of the features in human embryonic stem cells and neural progenitor cells awaits experimental validation, our results from epiNet will give us a hint to design future studies.

## Discussion

In this study, we built epiNet, a machine learning model to predict genome-wide CG methylation patterns of mammalian oocytes based on a limited number of other epigenetic features. epiNet captured the crosstalk between the epigenetic features and found that a combination of H3K36me3 and H3K4me3 can predict the CG methylation patterns of mouse oocytes quite accurately. Are the two histone modifications biologically relevant to CG methylation? DNMT3A, an enzyme responsible for the de novo CG methylation in oocytes, contains a PWWP domain, which recognizes H3K36me3, and an ADD domain, which recognizes H3K4me0 (regions devoid of H3K4me3) [4, 5]. Previous gene knockout (KO) studies showed that the depletion of H3K36me3 in oocytes causes a genome-wide loss of CG methylation (with occasional local gains) [6] and that the depletion of H3K4me3 causes local changes in CG methylation (more losses than gains) [12]. These results suggest that the two features are indeed biologically relevant. This in turn suggests that prediction using epiNet may aid experimental biologists in tasks such as the selection of KO targets among the epigenetic modification enzyme genes. The epiNet model is also applicable to cross-species prediction of the CG methylation pattern in human oocytes, although the predictive power seems somewhat lower.

The CG methylation establishment in mouse oocytes has been viewed as a transcription-coupled event, based on the previous studies on individual loci [28] and the entire genome [16]. While the CG methylation level is generally high in transcribed regions, our results suggest that the transcriptome data is not directly associated with the CG methylation pattern in mouse oocytes (Fig. 1b, d). Rather, a transcription-coupled histone modification, H3K36me3, is the major contributor to the CG methylation prediction in oocytes. This is consistent with the recent report that transcription likely impacts CG methylation through histone modifications and chromatin remodeling in mouse oocytes [29].

Lastly, we confirmed that epiNet is applicable to mammalian cells other than oocytes, such as embryonic stem cells and neuronal progenitor cells, the outcome of which suggested that different histone modifications play dominant roles in the formation of the CG methylation pattern in different cell types. The biological relevance of the prediction was confirmed by the gene KO studies in mouse embryonic stem cells [26, 27]. While the biological relevance in other cell types awaits experimental validation, epiNet should help to design biological experiments in future studies.

## Conclusions

The epiNet models could predict the CG methylation patterns of mammalian oocytes and embryonic stem cells accurately based on datasets from a limited number of other epigenetic features and could infer the crosstalk between the features and CG methylation. The available gene KO data suggest that the crosstalk inferred by epiNet is indeed biologically relevant. It also has the advantage of cross-species application. Our findings provide insight into the epigenetic crosstalk in mammalian oocytes and embryonic stem cells and demonstrate the usefulness of machine learning approaches in designing or complementing biological experiments in epigenetics studies.

## Methods

### Data processing

The datasets used in this study are summarized in Additional file 1: Table S1. The sequence reads were trimmed to remove low quality bases and adaptor sequences using Trim Galore 0.3.3 [30]. To obtain a CG methylation pattern, WGBS reads of mouse oocytes were mapped to mouse genome mm10 using Bismark 0.20.0 [31]. The WGBS data of human oocytes were downloaded from the NBDC human database [19]. The methylation levels of CG sites covered by 5–100 reads were extracted for downstream analyses. Bins with less than five informative CG sites were excluded. To obtain a histone modification or a chromatin accessibility pattern, ChIP-seq or CUT&RUN reads (for histone modification) or DNase-seq or ATAC-seq reads (for chromatin accessibility) of mouse and human oocytes were mapped to mm10 or human genome hg19 by bowtie2 2.2.9 [32]. Duplicate and low-quality reads (MapQ < 5) were removed using Picard 2.6.0 [33]. To obtain a transcription profile, RNA-seq reads of mouse oocytes were mapped to mm10 by HISAT2 2.0.5 [34]. The WGBS and histone modification data of human cell types (other than oocytes) were downloaded from the NIH Roadmap Epigenomics database [35]. In order to be used by epiNet, CG methylation levels (between 0 and 100%) were scaled between 0 and 1. FPKM values of other features were scaled between 0 and 1 by assigning the 95th percentile value as 0.95.

### The structure and application of epiNet

The model predicts CG methylation levels of bins from FPKM values of epigenetic features. It was implemented on python 3.6.8 [36], Tensorflow 1.14.0 [37] and Keras 2.2.4 [38]. The model consisted of four layers, including one convolutional layer and one fully connected layer. The genome was divided into 1-kb or 50-kb bins. Scaled CG methylation levels and FPKM values of input features were calculated for these bins. For each bin with *N* features, we constructed an *N* × 2 feature matrix *s* where *s*_i1_ is the FPKM value of the *i*th feature in the bin and *s*_i2_ is the mean FPKM value of the *i*th feature from 10 nearby bins. The input matrix *s* is first transformed by a two-dimensional convolutional layer with a kernel size of 2 for each feature, followed by the rectified linear unit (ReLU) function. The output, *X*_*fi*_, at the filter *f* (total 64 filters) for the *i*th feature is represented as follows:

1 $$X_{{fi}} = {\text{ReLU}}\left( {\mathop \sum \limits_{{k = 1}}^{2} \left( {w_{{fk}} s_{{ik}} } \right)} \right)$$

where *w*_*fk*_ is the weight of convolutional filter *f* of kth element of input matrix *s*_i_ of the *i*th feature. All outputs in the previous layer are compiled into a single *N* × 64 matrix. This single matrix is flattened to a 64 N-dimensional vector *Y*. *Y* is transformed to a 256-dimensional vector *Z*:

2 $$Z={\mathrm{ReLU}}\left({W}^{(2)}\cdot Y+{b}^{(2)}\right)$$

where *W*^(2)^ and *b*^(2)^ are the 256 × 64 N weight matrix and 1 × 64 N bias matrix, respectively. The final output layer transforms *Z* to predicted CG methylation level $\widehat{C}$:

3 $$\widehat{C}={\mathrm{ReLU}}\left({W}^{(3)}\cdot Z+{b}^{(3)}\right)$$

where *W*^(3)^ and *b*^(3)^ are a 1 × 256 weight matrix and 1 × 256 bias matrix respectively.

Otherwise noted, we used data from chromosome 1 as the test set, data from chromosomes 2 and 3 as the validation set, and data from all other chromosomes as the training set for both mouse and human. Training was performed by fitting the model on the training set with a batch size of 100 and by optimizing the hyperparameters through minimizing the mean squared error *F* in total *V* bins of the validation set, until there was no more reduction in *F* for 20 epochs:

4 $$F = \frac{1}{V}\mathop \sum \limits_{{v = 1}}^{V} \left( {C_{v} - \hat{C}_{v} } \right)^{2}$$

where ${C}_{v}$ and ${\widehat{C}}_{v}$ are the actual and predicted CG methylation levels of the *v*th bin respectively. The final model performance was evaluated on the test dataset, by calculating the Pearson correlation coefficient between the actual and predicted CG methylation patterns.

### Linear regression

Linear regression was performed using class LinearRegression of scikit-learn 0.23.1 [39] on python 3.6.8 [36].

### Data visualization and statistical analysis

Genome browser shots were generated using Integrative Genomics Viewer [40]. Pearson correlation coefficients between the eight input features and CG methylation were determined by deepTools 3.3.1 [41].

## Supplementary Information

**Additional file 1: Fig. S1.** Incorporation of the mean FPKM value of the neighboring region around the bin as input. **Fig. S2.** The CG methylation patterns of mouse FGOs depleted of H3K36me3 or H3K4me3. **Table S1.** Data used in this study. **Table S2.** Performance of epiNet based on actual versus randomly shuffled data. **Table S3.** Pearson correlation of histone modfications with CG methylation in cell types other than oocytes.

## Data Availability

The data used in this study are summarized in Additional file 1: Table S1. The epiNet model is available through GitHub at https://github.com/donalday/epiNet under the GNU General Public License v3 [42].

## Abbreviations

ATAC-seq: Assay for transposase-accessible chromatin with high-throughput sequencing
ChIP-seq: Chromatin immunoprecipitation with high-throughput sequencing
chr: Chromosome
CUT&RUN: Cleavage under targets and release using nuclease
DNase-seq: DNase I hypersensitive sites sequencing
ES cell: Embryonic stem cell
FGO: Fully-grown mouse oocyte
FPKM: Fragment per kb per million mapped reads
kb: Kilobase
KO: Knockout
NP cell: Neuronal progenitor cell
RefSeq: Reference sequence
ReLU: Rectified linear unit
RNA-seq: RNA sequencing
WGBS: Whole genome bisulfite sequencing

## References

[1] Allis CD, Jenuwein T (2016). The molecular hallmarks of epigenetic control. Nat Rev Genet.

[2] Soshnev AA, Josefowicz SZ, Allis CD (2016). Greater than the sum of parts: complexity of the dynamic epigenome. Mol Cell.

[3] Sendžikaitė G, Kelsey G (2019). The role and mechanisms of DNA methylation in the oocyte. Essays Biochem.

[4] Rondelet G, Maso TD, Willems L, Wouters J (2016). Structural basis for recognition of histone H3K36me3 nucleosome by human de novo DNA methyltransferases 3A and 3B. J Struct Biol.

[5] Otani J, Nankumo T, Arita K, Inamoto S, Ariyoshi M, Shirakawa M (2009). Structural basis for recognition of H3K4 methylation status by the DNA methyltransferase 3A ATRX–DNMT3–DNMT3L domain. Embo Rep.

[6] Xu Q, Xiang Y, Wang Q, Wang L, Brind’Amour J, Bogutz AB (2019). SETD2 regulates the maternal epigenome, genomic imprinting and embryonic development. Nat Genet.

[7] Lu L, Lin K, Qian Z, Li H, Cai Y, Li Y (2010). Predicting DNA methylation status using word composition. J Biomed Sci Eng.

[8] Zheng H, Wu H, Li J, Jiang S-W (2013). CpGIMethPred: computational model for predicting methylation status of CpG islands in human genome. BMC Med Genomics.

[9] Angermueller C, Lee HJ, Reik W, Stegle O (2017). DeepCpG: accurate prediction of single-cell DNA methylation states using deep learning. Genome Biol.

[10] Tian Q, Zou J, Tang J, Fang Y, Yu Z, Fan S (2019). MRCNN: a deep learning model for regression of genome-wide DNA methylation. BMC Genomics.

[11] Hanna CW, Taudt A, Huang J, Gahurova L, Kranz A, Andrews S (2018). MLL2 conveys transcription-independent H3K4 trimethylation in oocytes. Nat Struct Mol Biol.

[12] Shirane K, Toh H, Kobayashi H, Miura F, Chiba H, Ito T (2013). Mouse oocyte methylomes at base resolution reveal genome-wide accumulation of non-CpG methylation and role of DNA methyltransferases. PLoS Genet.

[13] Zheng H, Huang B, Zhang B, Xiang Y, Du Z, Xu Q (2016). Resetting epigenetic memory by reprogramming of histone modifications in mammals. Mol Cell.

[14] Wang C, Liu X, Gao Y, Yang L, Li C, Liu W (2018). Reprogramming of H3K9me3-dependent heterochromatin during mammalian embryo development. Nat Cell Biol.

[15] Au Yeung WK, Brind’Amour J, Hatano Y, Yamagata K, Feil R, Lorincz MC (2019). Histone H3K9 methyltransferase G9a in oocytes is essential for preimplantation development but dispensable for CG methylation protection. Cell Rep.

[16] Veselovska L, Smallwood SA, Saadeh H, Stewart KR, Krueger F, Maupetit-Méhouas S (2015). Deep sequencing and de novo assembly of the mouse oocyte transcriptome define the contribution of transcription to the DNA methylation landscape. Genome Biol.

[17] Inoue A, Jiang L, Lu F, Suzuki T, Zhang Y (2017). Maternal H3K27me3 controls DNA methylation-independent imprinting. Nature.

[18] Smallwood SA, Tomizawa S, Krueger F, Ruf N, Carli N, Segonds-Pichon A (2011). Dynamic CpG island methylation landscape in oocytes and preimplantation embryos. Nat Genet.

[19] Okae H, Chiba H, Hiura H, Hamada H, Sato A, Utsunomiya T (2014). Genome-wide analysis of DNA methylation dynamics during early human development. PLoS Genet.

[20] Xia W, Xu J, Yu G, Yao G, Xu K, Ma X (2019). Resetting histone modifications during human parental-to-zygotic transition. Science.

[21] Chen T, Ueda Y, Dodge JE, Wang Z, Li E (2003). Establishment and maintenance of genomic methylation patterns in mouse embryonic stem cells by Dnmt3a and Dnmt3b. Mol Cell Biol.

[22] Ziller MJ, Ortega JA, Quinlan KA, Santos DP, Gu H, Martin EJ (2018). Dissecting the functional consequences of de novo DNA methylation dynamics in human motor neuron differentiation and physiology. Cell Stem Cell.

[23] Habibi E, Brinkman AB, Arand J, Kroeze LI, Kerstens HHD, Matarese F (2013). Whole-genome bisulfite sequencing of two distinct interconvertible DNA methylomes of mouse embryonic stem cells. Cell Stem Cell.

[24] Zhang B, Zheng H, Huang B, Li W, Xiang Y, Peng X (2016). Allelic reprogramming of the histone modification H3K4me3 in early mammalian development. Nature.

[25] Yue F, Cheng Y, Breschi A, Vierstra J, Wu W, Ryba T (2014). A comparative encyclopedia of DNA elements in the mouse genome. Nature.

[26] Douillet D, Sze CC, Ryan C, Piunti A, Shah AP, Ugarenko M (2020). Uncoupling histone H3K4 trimethylation from developmental gene expression via an equilibrium of COMPASS, Polycomb and DNA methylation. Nat Genet.

[27] Li Y, Zheng H, Wang Q, Zhou C, Wei L, Liu X (2018). Genome-wide analyses reveal a role of Polycomb in promoting hypomethylation of DNA methylation valleys. Genome Biol.

[28] Chotalia M, Smallwood SA, Ruf N, Dawson C, Lucifero D, Frontera M (2009). Transcription is required for establishment of germline methylation marks at imprinted genes. Gene Dev.

[29] Gahurova L, Tomizawa S, Smallwood SA, Stewart-Morgan KR, Saadeh H, Kim J (2017). Transcription and chromatin determinants of de novo DNA methylation timing in oocytes. Epigenet Chromatin.

[30] Krueger F. Trim Galore. Github. https://github.com/FelixKrueger/TrimGalore.

[31] Krueger F, Andrews SR (2011). Bismark: a flexible aligner and methylation caller for bisulfite-seq applications. Bioinformatics.

[32] Langmead B, Salzberg SL (2012). Fast gapped-read alignment with Bowtie 2. Nat Methods.

[33] Picard. http://broadinstitute.github.io/picard. Accessed 25 May 2021.

[34] Kim D, Landmead B, Salzberg SL (2015). HISAT: a fast spliced aligner with low memory requirements. Nat Methods.

[35] Kundaje A, Meuleman W, Ernst J, Bilenky M, Yen A, Heravi-Moussavi A (2015). Integrative analysis of 111 reference human epigenomes. Nature.

[36] van Rossum G, de Boer J (1991). Interactively testing remote servers using the Python programming language. CWI Q.

[37] Abadi M, Agarwal A, Barham P, Brevdo E, Chen Z, Citro C, et al. TensorFlow: a system for large-scale machine learning. https://www.tensorflow.org.

[38] Chollet F. Keras. Github. https://github.com/keras-team/keras.

[39] Pedregosa F, Varoquaux G, Gramfort A, Michel V, Thirion B, Grisel O (2011). Scikit-learn: machine learning in Python. J Mach Learn Res.

[40] Robinson J, Thorvaldsdóttir H, Winckler W, Guttman M, Lander ES, Getz G (2011). Integrative genomics viewer. Nat Biotechnol.

[41] Ramírez F, Ryan DP, Grüning B, Bhardwaj V, Kilpert F, Richter AS (2016). deepTools2: a next generation web server for deep-sequencing data analysis. Nucleic Acids Res.

[42] Au Yeung WK. epiNet. Github. https://github.com/donalday/epiNet.

