# Supplementary material for: A convolutional neural network-based regression model to infer the epigenetic crosstalk responsible for CG methylation patterns
Source: BMC Bioinformatics. 2021 Jun 23;22:341. doi: 10.1186/s12859-021-04272-8 (PMC8220828; doi:10.1186/s12859-021-04272-8)
Supplement: Supplementary file 1 — Additional file 1: Fig. S1. Incorporation of the mean FPKM value of the neighboring region around the bin as input. Fig. S2. The CG methylation patterns of mouse FGOs depleted of H3K36me3 or H3K4me3. Table S1. Data used in this study. Table S2. Performance of epiNet based on actual versus randomly shuffled data. Table S3. Pearson correlation of histone modfications with CG methylation in cell types other than oocytes. [file 12859_2021_4272_MOESM1_ESM.pdf]

Supplementary information of "A convolutional neural network-based regression model to infer the epigenetic crosstalk responsible for CG methylation patterns"

Additional file 1: Fig. S1-2, Tables S1-3.

Fig. S1

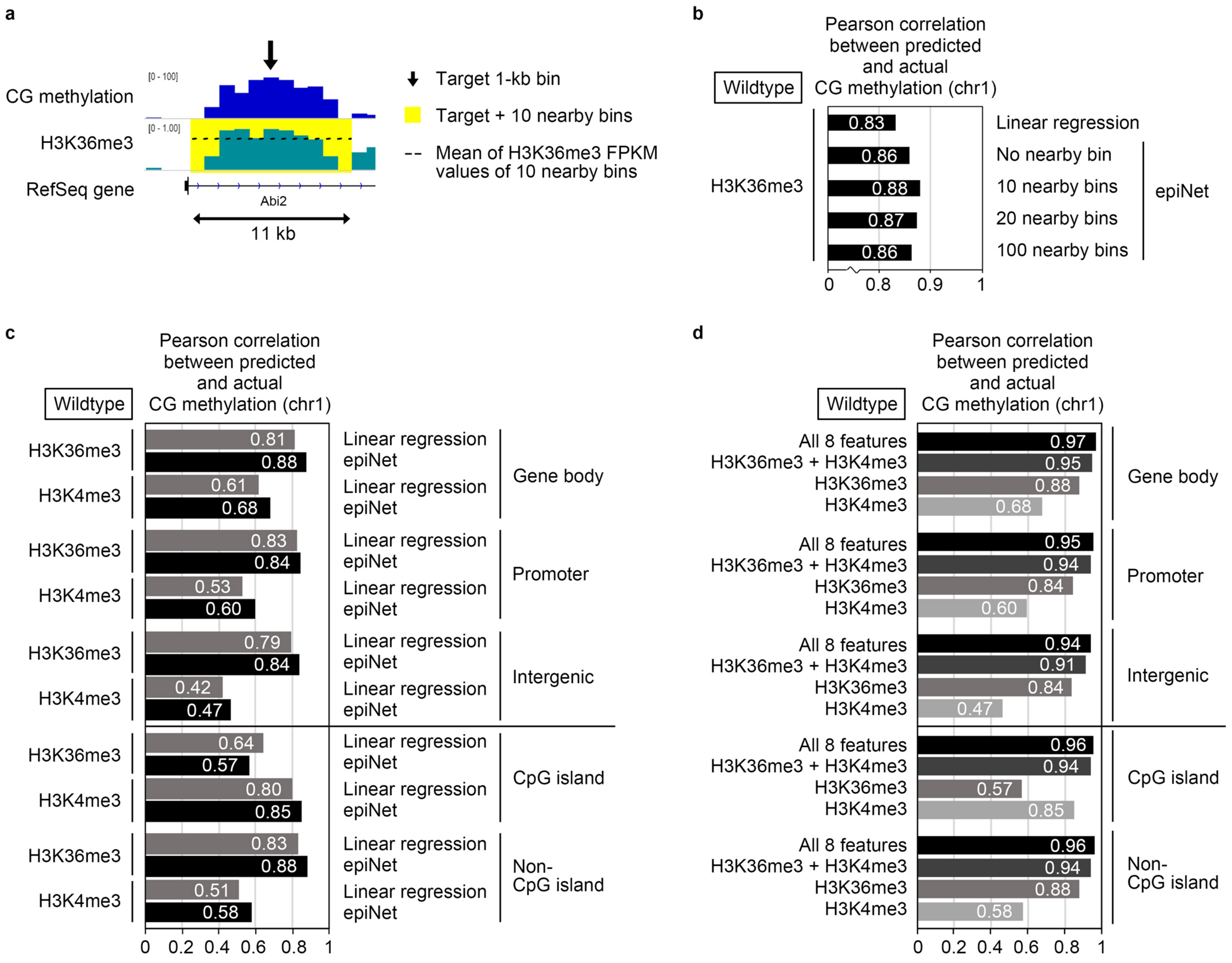
a
CG methylation
H3K36me3
RefSeq gene
[0 - 100]
[0 - 1.00]
Abi2
11 kb
Target 1-kb bin
Target + 10 nearby bins
Mean of H3K36me3 FPKM values of 10 nearby bins
b
Wildtype
Pearson correlation between predicted and actual CG methylation (chr1)
H3K36me3
0.83
0.86
0.88
0.87
0.86
Linear regression
No nearby bin
10 nearby bins
20 nearby bins
100 nearby bins
epiNet
0
0.8
0.9
1
c
Wildtype
Pearson correlation between predicted and actual CG methylation (chr1)
H3K36me3
H3K4me3
0.81
0.88
0.61
0.68
0.83
0.84
0.53
0.60
0.79
0.84
0.42
0.47
0.64
0.57
0.80
0.85
0.83
0.88
0.51
0.58
Linear regression
epiNet
Gene body
Promoter
Intergenic
CpG island
Non-CpG island
0
0.2
0.4
0.6
0.8
1
d
Wildtype
Pearson correlation between predicted and actual CG methylation (chr1)
All 8 features
H3K36me3 + H3K4me3
H3K36me3
H3K4me3
0.97
0.95
0.88
0.68
0.95
0.94
0.84
0.60
0.94
0.91
0.84
0.47
0.96
0.94
0.57
0.85
0.96
0.94
0.88
0.58
Gene body
Promoter
Intergenic
CpG island
Non-CpG island
0
0.2
0.4
0.6
0.8
1

Fig. S1 | Incorporation of the mean FPKM value of the neighboring region around the bin as input. a, A schematic representation of a genomic region containing the target bin in the middle. Both the FPKM value of the target bin and the mean of FPKM values of 10 nearby bins were used. **b**, Prediction of the CG methylation pattern by linear regression or epiNet, based on the H3K36me3 data. epiNet models with varying numbers of nearby bins (0, 10, 20 and 100) were tested. Pearson correlation coefficients between the predicted and actual CG methylation patterns are shown. **c**, The prediction of the CG methylation pattern at different genomic features by linear regression or epiNet based on the H3K36me3 and H3K4me3 data. All 1-kb bins of chromosome 1 were classified into those containing the gene body, the promoter (a 1-kb region upstream of the transcription start site), or neither of the two (intergenic), or those containing CpG island and no CpG island. Pearson correlation coefficients between the predicted and actual CG methylation patterns are shown. **d**, Prediction of the CG methylation pattern at different genomic features by epiNet based on all eight features, the H3K36me3 and H3K4me3 data, the H3K36me3 data, and the H3K4me3 data. The details are the same as in **c**.

Fig. S2

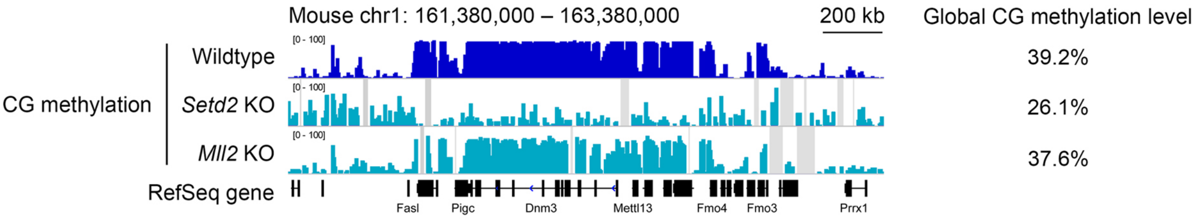

Mouse chr1: 161,380,000 – 163,380,000
200 kb
Global CG methylation level
CG methylation
Wildtype
[0 - 100]
39.2%
Setd2 KO
[0 - 100]
26.1%
Mll2 KO
[0 - 100]
37.6%
RefSeq gene
Fasl
Pigc
Dnm3
Mettl13
Fmo4
Fmo3
Prrx1

Fig. S2 | The CG methylation patterns of mouse FGOs depleted of H3K36me3 or H3K4me3. The WGBS data from published works [6,11] were reprocessed. The global CG methylation levels are indicated on the right. Gray boxes show regions without a sufficient number of reads.

Table S1 | Data used in this study.

Species	Feature	Cell type	Genotype	Accession number	Reference(s)
Mouse	CG methylation	FGO, embryonic stem cell	Wildtype, *Setd2* KO, *Mll2* KO	DRA000570, GSE112835, GSE93941, GSE41923	6, 11, 12, 40
	H3K36me3	FGO, embryonic stem cell	Wildtype, *Setd2* KO	GSE112835	6
	H3K9me3	Metaphase II oocyte, embryonic stem cell	Wildtype	GSE97778	14
	Transcription	FGO	Wildtype	GSE70116	16
	H3K27ac	FGO, embryonic stem cell	Wildtype, *Setd2* KO	GSE93941, GSE112835, GSE71434	6, 11, 41
	Chromatin accessibility	FGO	Wildtype	GSE92605	17
	H3K27me3	FGO, embryonic stem cell	Wildtype, *Setd2* KO	GSE76687, GSE112835, GSE49847	6, 13, 42
	H3K9me2	FGO	Wildtype	GSE112320	15
	H3K4me3	FGO, embryonic stem cell	Wildtype, *Setd2* KO	GSE93941, GSE112835	6, 11
Human	CG methylation	FGO, metaphase I oocyte, embryonic stem cell, neuronal progenitor cell	Not applicable	JGAS00000000006, GSE16256, GSE16368, GSE17312, GSE18927, GSE19465, GSE25246, GSE25247, GSE25248, GSE25249	19, 32
	H3K36me3	Embryonic stem cell, neuronal progenitor cell	Not applicable	GSE16256, GSE16368, GSE17312, GSE18927, GSE19465, GSE25246, GSE25247, GSE25248, GSE25249	32
	H3K9me3	Embryonic stem cell, neuronal progenitor cell	Not applicable	GSE16256, GSE16368, GSE17312, GSE18927, GSE19465, GSE25246, GSE25247, GSE25248, GSE25249	32
	H3K27ac	Embryonic stem cell, neuronal progenitor cell	Not applicable	GSE16256, GSE16368, GSE17312, GSE18927, GSE19465, GSE25246, GSE25247, GSE25248, GSE25249	32
	Chromatin accessibility	FGO	Not applicable	GSE124718	20
	H3K27me3	FGO, embryonic stem cell, neuronal progenitor cell	Not applicable	GSE124718, GSE16256, GSE16368, GSE17312, GSE18927, GSE19465, GSE25246, GSE25247, GSE25248, GSE25249	20, 32
	H3K4me3	FGO, embryonic stem cell, neuronal progenitor cell	Not applicable	GSE124718, GSE16256, GSE16368, GSE17312, GSE18927, GSE19465, GSE25246, GSE25247, GSE25248, GSE25249	20, 32

Table S2 | Performance of epiNet based on actual versus randomly shuffled data.

Pearson correlation coefficients are shown. Genomic coordinates of H3K36me3 and H3K4me3 FPKM values were randomly shuffled for comparison.

Feature	Perason correlation coefficient	
	Actual data	Shuffled data
H3K36me3	0.88	0.00
H3K4me3	0.58	0.00

Table S3 | Pearson correlation of histone modfications with CG methylation in cell types other than oocytes. The most correlated features for each cell type were indicated on the right.

	Pearson correlation with CG methylation					Most correlated feature
	H3K4me3	H3K9me3	H3K27ac	H3K27me3	H3K36me3	
Mouse embryonic stem cell	-0.74	0.01	-0.45	-0.29	-0.06	H3K4me3
Human embryonic stem cell	-0.71	-0.08	-0.37	-0.55	-0.19	H3K4me3
Human neuronal progenitor cell	-0.60	-0.09	-0.29	-0.24	-0.14	H3K4me3
